# LncRNA RP11-465B22.8 triggers esophageal cancer progression by targeting miR-765/KLK4 axis

**DOI:** 10.1038/s41420-021-00631-9

**Published:** 2021-09-24

**Authors:** Rui Hu, Rui Bi, Lianyong Jiang, Haibo Xiao, Xiao Xie, Hongtao Liu, Fengqing Hu

**Affiliations:** Department of Cardiothoracic Surgery Xinhua Hospital Affiliated to Shanghai Jiaotong University School of Medicine, Shanghai, 200092 China

**Keywords:** Tumour biomarkers, Cancer microenvironment

## Abstract

LncRNAs play an important role in tumorigenesis and progression; however, the function and mechanisms of lncRNAs in esophageal cancer (EC) remain largely unclear. In this study, we screened the differentially expressed lncRNAs in EC by using RNA-seq and one of the most upregulated lncRNAs, lncRNA RP11-465B22.8, was further characterized. LncRNA RP11-465B22.8 was upregulated in EC tissues and high lncRNA RP11-465B22.8 expression was associated with poor survival of EC patients. Ectopic expression of lncRNA RP11-465B22.8 enhanced the proliferation, migration, and invasion of EC cells, whereas knockdown of lncRNA RP11-465B22.8 led to the opposite effects. Mechanistically, lncRNA RP11-465B22.8 sponged miR-765 to increase the expression of KLK4. Moreover, LncRNA RP11-465B22.8 could be delivered from EC cells to macrophages via exosomes and subsequently induced M2 macrophage-induced cell migration and invasion. Our findings revealed a novel lncRNA RP11-465B22.8/miR-765/KLK4 pathway in EC and indicated that lncRNA RP11-465B22.8 might be a potential target for EC therapy.

## Introduction

Esophageal cancer (EC) is one of the most common gastrointestinal malignancies. In 2012, approximately 460,000 EC cases and 400,000 deaths occurred in the world [1]. Because of the unspecific symptoms, EC patients usually develop metastases at the time of diagnosis, which makes it difficult to therapy and leads to poor survival. Therefore, efforts are still needed to improve the treatment of EC, and understanding the mechanisms of EC development and progression is one of the important steps to achieve this goal.

Long noncoding RNAs (LncRNAs) are a class of endogenous cellular noncoding RNAs that have more than 200 nucleotides [2]. LncRNAs are essential in a number of biological processes, such as cell proliferation, migration, apoptosis, angiogenesis, inflammation, immune response, etc. [3, 4]. Aberration of lncRNAs has been reported to be associated with the pathogenesis of many diseases, such as aortic aneurysm, atherosclerosis, pathogen infection, as well as cancers [4–7]. For example, lncRNA AFAP1-AS1 has been found to be overexpressed in esophageal adenocarcinoma (EAC) and its silencing suppresses the proliferation, migration, and invasion of EAC cells [8]. Similarly, lncRNA HNF1A-AS1 is upregulated in primary EAC, and knockdown of HNF1A-AS1 represses cell proliferation, anchorage-independent growth, cell migration, and invasion [9].

Accumulating evidence has been demonstrated that lncRNAs trigger cancer progression via their interactions with different molecules, such as DNA, RNA, and proteins [10]. Acting as competing endogenous RNAs (ceRNAs) is one of the key mechanisms of lncRNAs to exert their functions, through which lncRNAs regulate target gene expression by competitively interacting with their common miRNAs [11]. For instance, KRTAP5-AS1 and lncRNA-TUBB2A sponge miR-596 and/or miR-3620-3p to increase the expression of Claudin-4, thus promoting the proliferation and invasion of gastric cancer cells [11]. LncRNA-ATB upregulates ZEB1 and ZEB2 by competitively binding the miR-200 family and induces EMT program and invasion in hepatocellular carcinoma cells [12]. However, the action and mechanisms of a majority of lncRNAs in EC remain unelucidated.

In this study, we screened lncRNAs that were differentially expressed in EC tissues by performing RNA-seq. LncRNA RP11-465B22.8, which was significantly upregulated in EC tissues was selected for further investigation. Our data showed that increased LncRNA RP11-465B22.8 expression was linked to shorter survival of patients with EC. LncRNA RP11-465B22.8 positively regulated KLK4 expression by sponging miR-765 and thus enhanced EC cell proliferation and migration both in vitro and in vivo. Moreover, lncRNA RP11-465B22.8 could be secreted by EC cells via exosomes. Exosomal lncRNA RP11-465B22.8 promoted M2 macrophage polarization, which in turn triggered EC cell migration and invasion. Our results provide evidence that lncRNA RP11-465B22.8 acts as an oncogene in EC, which may serve as a novel target for EC therapy.

## Results

### LncRNA RP11-465B22.8 expression is increased in EC tissues and linked to poor survival

**Fig. 1:**
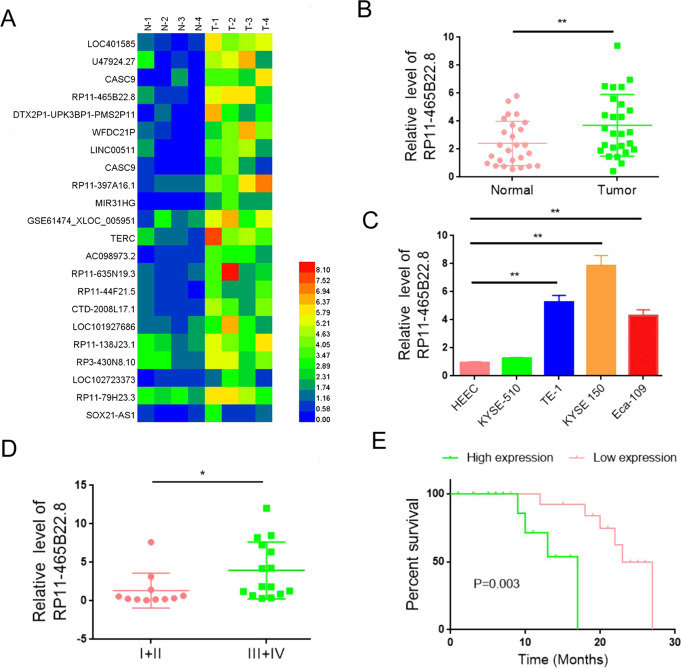
LncRNA RP11-465B22.8 is upregulated in EC tissues and cells. **A** Heatmap representing the top differently expressed lncRNAs in EC tissues (*n* = 4). Green shows high expression and blue shows low expression. **B** qRT-PCR analysis of the expression of lncRNA RP11-465B22.8 in EC tissues. **C** qRT-PCR analysis of the expression of lncRNA RP11-465B22.8 in EC cells. **D** qRT-PCR analysis of lncRNA RP11-465B22.8 in EC tumors at different states. **E** High lncRNA RP11-465B22.8 expression predicted poor survival of EC patients. *P < 0.05, **P < 0.01.

To identify the key lncRNAs involved in EC progression, we performed RNA-seq analysis using three pairs of EC tissues and their matched adjacent normal tissues. A total of 151 lncRNAs were upregulated and 104 lncRNAs were downregulated in tumor samples compared with normal samples (Fig. 1A). Among those differentially expressed lncRNAs, we mainly focused on lncRNA RP11-465B22.8, which is one of the most upregulated lncRNAs and its function is not yet understood. Consistently, quantitative real-time polymerase chain reaction (qRT-PCR) assay showed that the expression of LncRP11-465B22.8 was markedly increased in esophageal tumors compared with normal tissues (Fig. 1B). We also examined lncRNA RP11-465B22.8 expression in several EC cell lines and found that the expression of lncRNA RP11-465B22.8 was higher in three EC cell lines (TE-1, Eca-109, and KYSE-150), compared with the normal endometrial epithelial cell line (HEEC) (Fig. 1C). Moreover, survival analysis revealed that high lncRNA RP11-465B22.8 expression was associated with higher tumor stage and poor survival of patients with EC (Fig. 1D, E, Supplementary Table 2). Together, these results indicate that lncRP11-465B22.8 is a biomarker of poor prognosis in EC patients.

### LncRNA RP11-465B22.8 promotes esophageal tumorigenesis

**Fig. 2:**
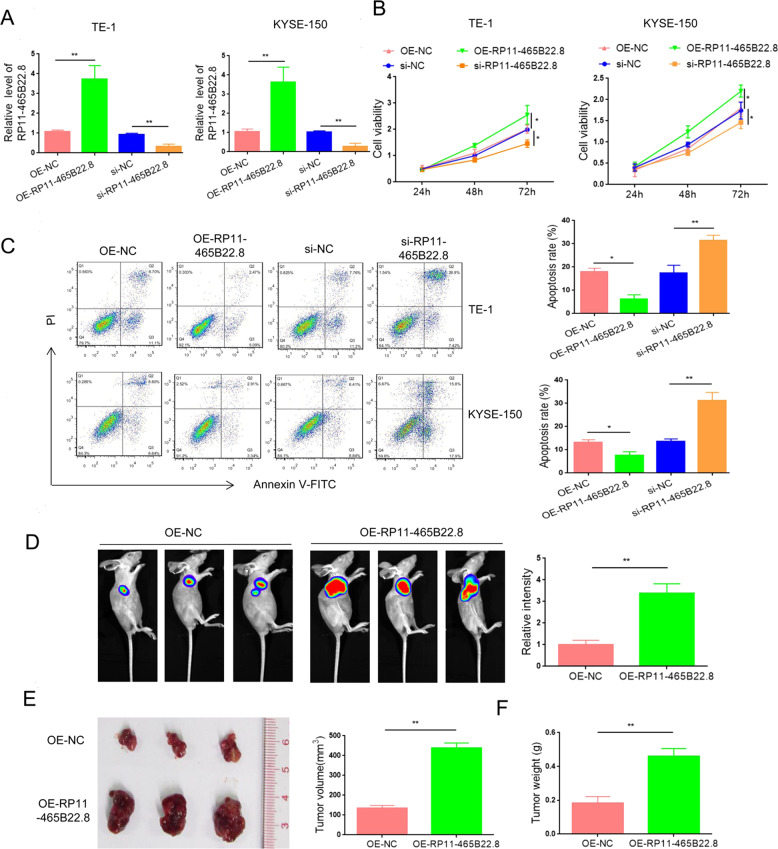
LncRNA RP11-465B22.8 enhances EC tumorigenesis. **A** qRT-PCR analysis of lncRNA RP11-465B22.8 expression in EC cells after lncRNA RP11-465B22.8 overexpression or knockdown. **B** CCK-8 assay analysis of the viability of EC cells after lncRNA RP11-465B22.8 transfection or silencing. **C** Apoptosis analysis of EC cells following lncRNA RP11-465B22.8 overexpression or depletion. **D** Luciferase intensity in mice after injection with TE-1 cells expressing lncRNA RP11-465B22.8 or control vector (*n* = 5). **E** Representative images of tumors isolated from mice injected with lncRNA RP11-465B22.8-overexpressed TE-1 cells or control cells (*n* = 5). **F** Tumor weight of tumors formed by TE-1 cells overexpressing lncRNA RP11-465B22.8 or control vector. *P < 0.05, **P < 0.01.

To access the biological action of lncRNA RP11-465B22.8 in EC, we overexpressed or silenced lncRNA RP11-465B22.8 in EC cells (TE-1, KYSE-150, and KYSE-150). The efficiency of lncRNA RP11-465B22.8 overexpression or knockdown was verified by qRT-PCR (Fig. 2A and Fig. S1). We then detected the influence of lncRNA RP11-465B22.8 on EC cell proliferation. After overexpression of lncRNA RP11-465B22.8, the viability was significantly increased in TE-1 and KYSE-150 cells (Fig. 2B). In contrast, the proliferation was remarkably decreased in TE-1 and KYSE-150 cells upon lncRNA RP11-465B22.8 silencing (Fig. 2B). Flow cytometry analysis showed that transfection of lncRNA RP11-465B22.8 reduced the apoptosis rate in both TE-1 and KYSE-150 cells, whereas knockdown of RP11-465B22.8 resulted in the opposite effect (Fig. 2C). In agreement with these results, the expressions of cleaved caspase 3 and Bcl-2 were increased while Bax expression was decreased in RP11-465B22.8-overexpressed EC cells (Fig. S2). On the contrary, the levels of cleaved caspase 3 and Bcl-2 were decreased and the Bax level was increased in RP11-465B22.8-knockdown cells (Fig. S2). Furthermore, the effect of lncRNA RP11-465B22.8 on tumor growth in vivo was accessed by using a xenograft tumor assay. Luciferase-labeled TE-1 cells stably overexpressing lncRNA RP11-465B22.8 or control vector were subcutaneously injected into nude mice and the luciferase intensity was monitored 30 days after inoculation. The results showed that ectopic expression of lncRNA RP11-465B22.8 remarkably increased the luciferase intensity in mice (Fig. 2D). Consistently, larger tumor volume and greater tumor weight were observed in the extracted tumors formed by TE-1/RP11-465B22.8 cells compared with those formed by the control cells (Fig. 2E, F). Collectively, these data suggest that lncRNA RP11-465B22.8 has an oncogenic role in EC.

### LncRNA RP11-465B22.8 triggers EC metastasis

**Fig. 3:**
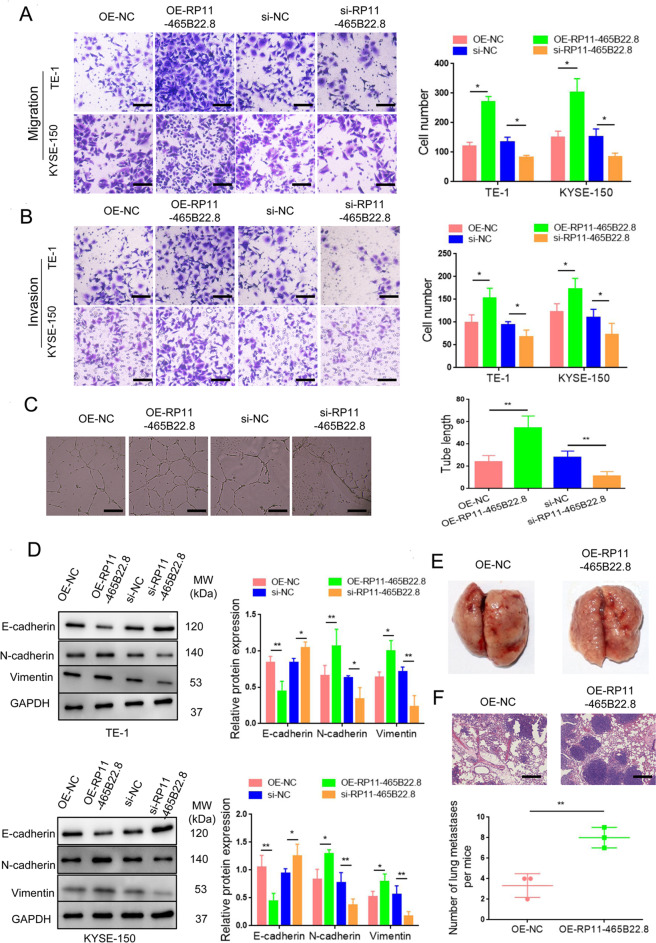
LncRNA RP11-465B22.8 promotes EC cell migration, invasion, and metastasis. **A**, **B** The migration and invasion in EC cells transfected with lncRNA RP11-465B22.8-overexpressing plasmid or si-RP11-465B22.8. **C** Tube formation of HUVECs treated with media from EC cells transfected with lncRNA RP11-465B22.8-overexpressing plasmid or si-RP11-465B22.8. **D** The expression levels of epithelial (E-cadherin) and mesenchymal markers (N-cadherin and vimentin) in EC cells transfected with lncRNA RP11-465B22.8-overexpressing plasmid or si-RP11-465B22.8. **E** Representative images of lung tissues from mice injected with lncRNA RP11-465B22.8-overexpressed cells or control cells (*n* = 3). **F** H&E staining of lung sections from mice injected with lncRNA RP11-465B22.8-overexpressed cells or control cells (*n* = 3). *P < 0.05, **P < 0.01.

To investigate the effect of lncRNA RP11-465B22.8 on EC cell motility, Transwell migration and invasion assays were performed. Compared with the control cells, the migration and invasion were significantly increased in lncRNA RP11-465B22.8-overexpressed TE-1 and KYSE-150 cells (Fig. 3A, B). In contrast, the motility of TE-1 and KYSE-150 cells was markedly inhibited following lncRNA RP11-465B22.8 knockdown (Fig. 3A, B). The potential function of lncRNA RP11-465B22.8 in angiogenesis was also addressed. Conditioned media were collected from TE-1 cells with lncRNA RP11-465B22.8 overexpression or knockdown and applied to HUVECs. The results showed that the treatment with conditioned medium from TE-1/RP11-465B22.8 cells significantly increased the tube formation of HUVECs, whereas exposure of conditioned medium from TE-1/si-RP11-465B22.8 cells to HUVECs resulted in a predominant decrease in the tube number (Fig. 3C). It is known that EMT progression plays an important role in cancer metastasis, thus we determined whether lncRNA RP11-465B22.8 could affect the EMT program. Overexpression of lncRNA RP11-465B22.8 in TE-1 and KYSE-150 cells decreased the expression of the epithelial protein (E-cadherin) but increased the expression of mesenchymal proteins (N-cadherin and vimentin) (Fig. 3D). On the contrary, silencing of lncRNA RP11-465B22.8 resulted in the opposite results in TE-1 and KYSE-150 cells (Fig. 3D). To confirmed that lncRNA RP11-465B22.8 could promote cell migration in vivo, a metastasis assay was performed in nude mice via tail vein injection of TE-1 cells overexpressing lncRNA RP11-465B22.8 or the control vector. An increased number of metastasis nodules was observed in lung tissues of mice injected with TE-1/RP11-465B22.8 cells compared with mice injected with TE-1/Vector cells (Fig. 3E, F). Taken together, these results indicate that lncRNA RP11-465B22.8 can promote EC cell migration both in vitro and in vivo.

### LncRNA RP11-465B22.8 promotes EC progression by targeting miR-765

**Fig. 4:**
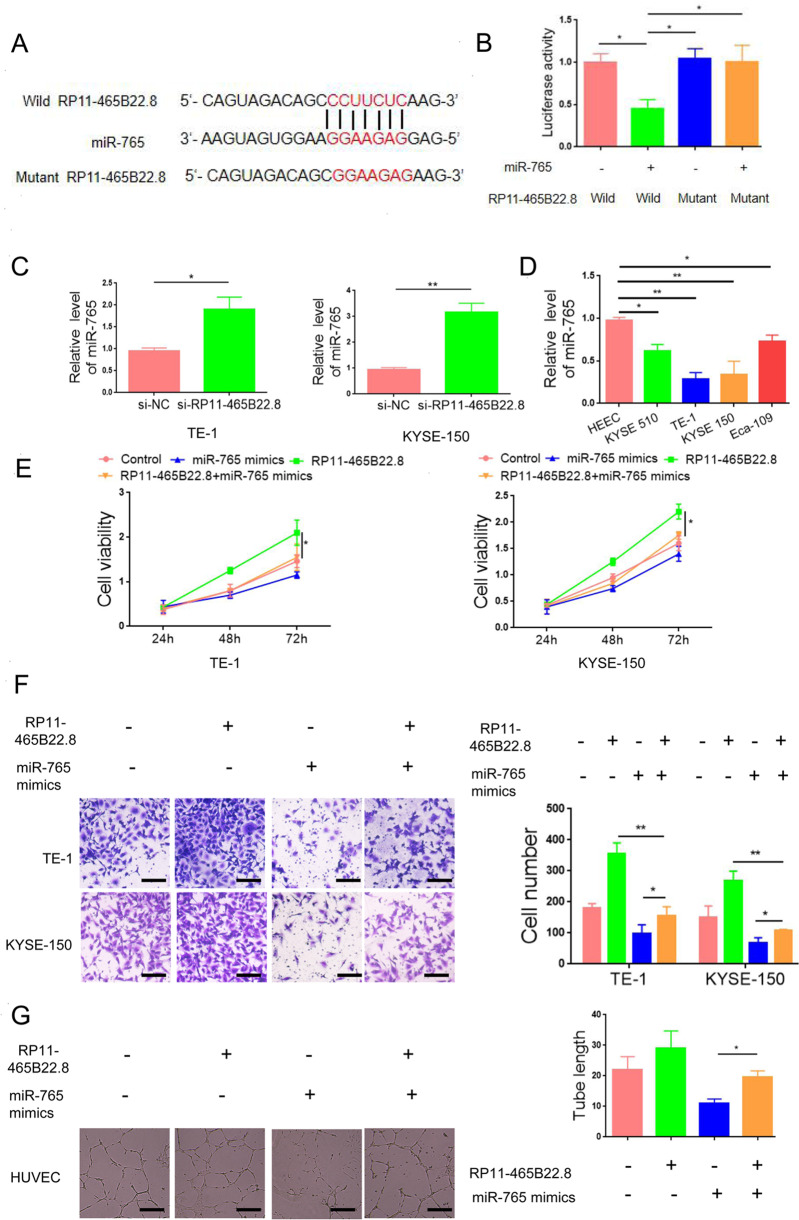
LncRNA RP11-465B22.8 promotes EC cell viability and mobility by sponging miR-765. **A** The predicted binding sites of miR-765 in lncRNA RP11-465B22.8. **B** Luciferase reporter assay in HEK293T cells transfected with miR-765 together with the wildtype (wild) or mutated lncRNA RP11-465B22.8 (mutant). **C** qRT-PCR analysis miR-765 expression in EC cells with lncRNA RP11-465B22.8 knockdown. **D** qRT-PCR analysis of miR-765 expression in different EC cell lines. **E** Transfection of miR-765 mimics abolished the enhanced effect of lncRNA RP11-465B22.8 on EC cell viability. **F** Transfection of miR-765 mimics abolished the enhanced effect of lncRNA RP11-465B22.8 on EC cell migration. **G** Transfection of miR-765 mimics abolished the promoting effect of conditioned medium from EC cells overexpressing lncRNA RP11-465B22.8 on the tube formation of HUVECs. *P < 0.05, **P < 0.01.

LncRNAs usually execute their function via targeting miRNAs. Using the ReqRNA platform, we identified miR-765 as a potential target of lncRNA RP11-465B22.8 (Fig. 4A), which has been reported to be associated with the prognosis in patients with esophageal squamous cell carcinoma [13]. To verify the direct interaction between miR-765 and lncRNA RP11-465B22.8, a luciferase reporter assay was performed. Transfection of miR-765 reduced the luciferase intensity of wild-type lncRNA RP11-465B22.8 but had no significant effect on lncRNA RP11-465B22.8 mutant (Fig. 4B). Knockdown of lncRNA RP11-465B22.8 elevated the expression of miR-765 in TE-1 and KYSE-150 cells (Fig. 4C). When examining the expression of miR-765 in EC cell lines, we found that miR-765 was downregulated in EC cells compared with normal cells (Fig. 4D), indicating an inhibitory activity of miR-765 in EC. As expected, overexpression of miR-765 suppressed the proliferation and migration and upregulated the level of E-cadherin in EC cells (Fig. S3-S5). Moreover, transfection of miR-765 mimics abolished the enhancing effects of lncRNA RP11-465B22.8 on cell proliferation, migration, and tube formation (Fig. 4E–G). Collectively, these data suggest that miR-765 is a direct target of lncRNA RP11-465B22.8, through which lncRNA RP11-465B22.8 promotes EC metastasis.

### LncRNA RP11-465B22.8 enhances KLK4 expression by targeting miR-765

**Fig. 5:**
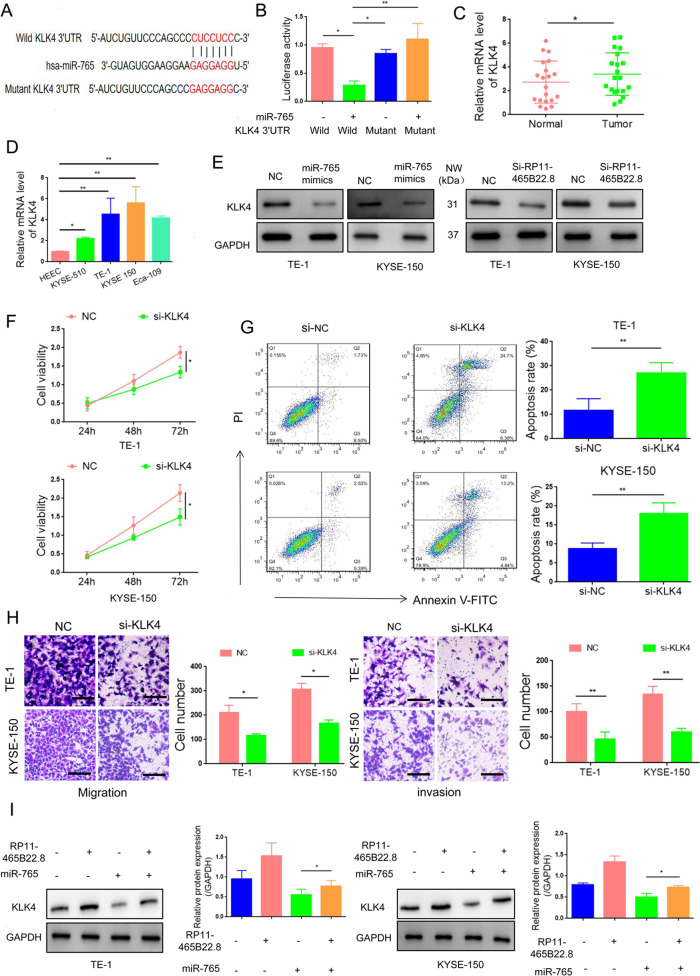
LncRNA RP11-465B22.8 promotes EC cell proliferation and migration by targeting miR-765/KLK4 axis. **A** The predicted binding sites of miR-765 in KLK4 3′UTR. **B** Luciferase reporter assay in HEK293T cells transfected with miR-765 together with the wildtype (wild) or mutated KLK4 3′UTR (Mutant). **C**, **D** qRT-PCR analysis of miR-765 expression in EC tissues (*n* = 20) and cell lines. **E** qRT-PCR analysis of KLK4 expression in EC cells transfected with miR-765 mimics. **F** qRT-PCR analysis of KLK4 expression in EC cells transfected with lncRNA RP11-465B22.8. **G** The cell viability of EC cells transfected with si-KLK4. **H** The apoptosis rate of EC cells after KLK4 silencing. **I** The migration ability of EC cells with KLK4 knockdown. **J** Transfection with miR-765 blocked the promoting effect of lncRNA RP11-465B22.8 on KLK4 expression. *P < 0.05, **P < 0.01.

MiRNAs commonly function via the downregulation of target proteins. Therefore, we used TagetScan, miRDB, and miRcode platforms to search the potential targets of miR-765 and found KLK4 as a candidate (Fig. 5A), which has a critical effect on cancer progression [14–16]. Luciferase assay showed that transfection with miR-765 mimics decreased the luciferase activity of wild-type KLK4 3′-UTR, but did not alter the activity of KLK4 3′-UTR mutant (Fig. 5B), indicating a direct interaction between miR-765 and KLK4. KLK4 expression was significantly increased in esophageal tumors and cells compared with controls (Fig. 5C, D). Treatment with miR-765 mimics greatly decreased the expression of KLK4, which was consistent with the effect by silencing of lncRNA RP11-465B22.8 (Fig. 5E, F). Moreover, silencing of KLK4 expression decreased the proliferation and migration and increased apoptosis in EC cells (Fig. 5G, H), suggesting that KLK4 functions as a tumor promoter in EC. In addition, transfection of miR-765 mimics reduced the enhancement in KLK4 expression induced by lncRNA RP11-465B22.8 (Fig. 5I). Together, these results indicate that lncRNA RP11-465B22.8 positively regulated KLK4 expression via targeting miR-765, thus contributing to EC progression.

### Exosomal lncRNA RP11-465B22.8 triggers M2 macrophage-mediated cell migration and invasion

**Fig. 6:**
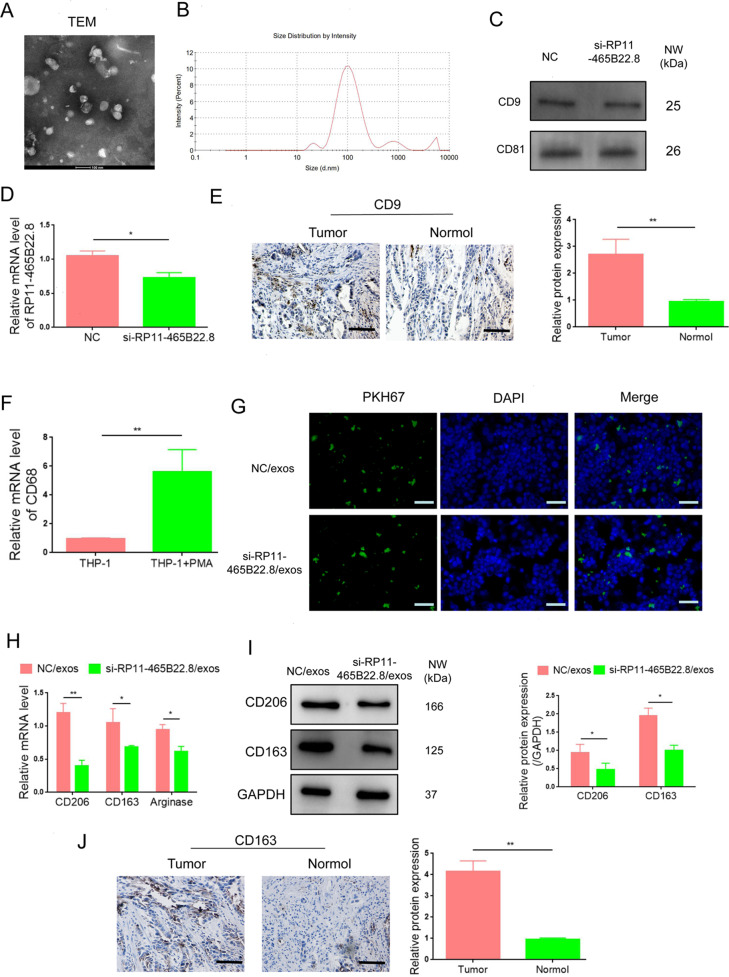
Exosomal lncRNA RP11-465B22.8 promotes M2 polarization. **A** TEM analysis of isolated exosomes from KYSE-150 cells with lncRNA RP11-465B22.8 knockdown. **B** The size distribution of exosomes from KYSE-150 cells with lncRNA RP11-465B22.8 silencing. **C** Western blot analysis of the expression of CD9 and CD61 in exosomes from lncRNA RP11-465B22.8-silenced KYSE-150 cells. **D** qRT-PCR analysis of lncRNA RP11-465B22.8 in exosomes from KYSE-150 cells transfected with si-P11-465B22.8. **E** Immunochemistry staining of CD9 expression in EC tissues. **F** qRT-PCR analysis of CD68 in THP-1 cells with or without PMA treatment. **G** Immunofluorescence staining detecting the uptake of PKH67-labeled (green) exosomes by PMA-induced macrophages. DAPI was used to stain the nucleus. **H** qRT-PCR of the mRNA expression of CD206, CD163, and Arginase in PMA-treated THP-1 cells. **I** Western blot analysis of the protein expression of CD206, CD163 in PMA-treated THP-1 cells. **J** Immunochemistry staining of CD163 expression in EC tissues. *P < 0.05, **P < 0.01.

**Fig. 7:**
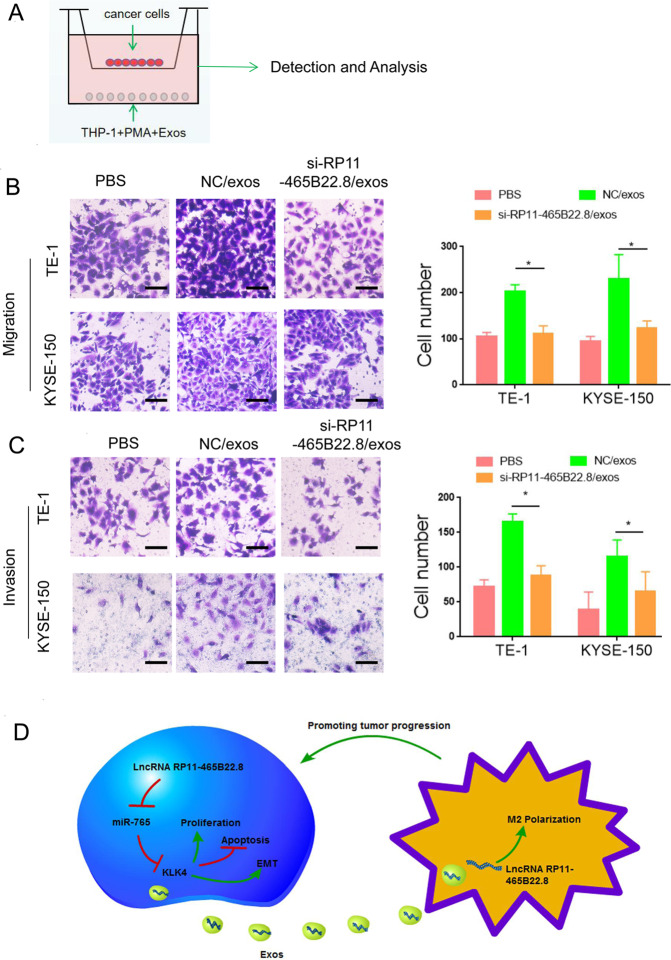
Exosomal lncRNA RP11-465B22.8 triggers M2 macrophage-mediated mobility in EC cells. **A** Schematic image indicating the coculture/migration system for macrophage migration analysis. **B**, **C** The migration and invasion abilities of PMA-induced macrophages after treatment with exosomes from KYSE-150 cells transfected with si-RP11-465B22.8 (si-RP11-465B22.8/exos) or control siRNA (NC/exos). **D** Schematic illustration of the mechanism by which lncRNA RP11-465B22.8 promotes EC progression. In EC cells, lncRNA RP11-465B22.8 elevates KLK4 expression by sponging miR-765, thus facilitating EC cell proliferation and migration. In addition, exosomal RP11-465B22.8 derived from EC cells promotes M2 polarization of macrophages, which further enhances EC progression.

The communication between cancer cells and immune cells in the tumor microenvironment is essential for cancer progression. Exosome-mediated delivery of lncRNAs to target cells is one of the most important mechanisms of cancer cells to regulate immune cells. M2 macrophages play a key role in cancer development. Thus, we investigated whether exosomal lncRNA RP11-465B22.8 had an effect on macrophage polarization. Exosomes were isolated from KYSE-150 cells following lncRNA RP11-465B22.8 knockdown and were confirmed by transmission electron microscopy (TEM) and Western blotting detection (Fig. 6A–C). The expression of lncRNA RP11-465B22.8 was decreased in exosomes derived from KYSE-150/si-RP11-465B22.8 cells (si-RP11-465B22.8/exos) compared with exosomes from the control cells (NC/exos) (Fig. 6D). Immunohistochemistry (IHC) analysis showed that the expression of exosome marker CD9 in esophageal tumor tissues was much higher than that in normal tissues (Fig. 6E), confirming the existence of exosomes in EC tissue. Human THP-1 cells were differentiated into macrophages by the treatment with phorbol 12-myristate 13-acetate (PMA). After exposure to PMA, increased CD68 expression was observed in THP-1 cells (Fig. 6F), indicating a successful induction of macrophages. We then examined whether cancer-derived exosomes could be taken up by macrophages. Exosomes derived from KYSE-150 transfected with si-RP11-465B22.8 (si-RP11-465B22.8/exos) or control siRNA (NC/exos) were stained with PKH67 and incubated with PMA-induced macrophages. A number of PKH67 green spots were visualized in macrophages (Fig. 6G). Moreover, treatment with si-RP11-465B22.8/exos resulted in decreased expressions in M2 markers (CD206 and CD163) and Arginase (Fig. 6H, I), indicating an impairment in M2 polarization of macrophages. In addition, compared with normal tissues, the expression of CD163 was significantly increased in EC tissues (Fig. 6J), indicating a greater population of M2 macrophages in esophageal tumors. To further explore the function of M2 macrophages induced by exosomal lncRNA RP11-465B22.8, an in vitro coculture system with macrophages and EC was utilized (Fig. 7A). The coculture/migration assay showed that M2 macrophages treated with si-RP11-465B22.8/exos remarkably decreased the migration of TE-1 and KYSE-150 cells (Fig. 7B). Similarly, the invasion ability of TE-1 and KYSE-150 cells was significantly inhibited by si-RP11-465B22.8/exos-exposed M2 macrophages (Fig. 7C). Collectively, our results show that exosomal lncRNA RP11-465B22.8 promotes M2 polarization of macrophages, which contributes to EC cell migration.

## Discussion

Although the treatment of EC has been improved, the therapeutic efficacy is unsatisfactory and metastasis remains a major threat. The progression of cancer metastasis is complicated, which involves a number of cellular molecules, including lncRNAs. The significance of lncRNAs in cancer development and progression has been documented; however, the function and mechanism of a majority of lncRNAs are not fully understood in EC. In this study, we investigated the biological activity of an uncharacterized lncRNA RP11-465B22.8 in EC. Our data revealed that lncRNA RP11-465B22.8 was upregulated in EC tissues and its high expression predicted poor survival in EC patients. Functional studies showed that lncRNA RP11-465B22.8 promotes the proliferation, migration, and invasion of EC cells in vitro. Moreover, lncRNA RP11-465B22.8 enhanced EC tumorigenesis and metastasis in vivo. Therefore, our results suggest that lncRNA RP11-465B22.8 acts as an oncogene in EC.

Serving as a ceRNA is one of the important mechanisms by which lncRNAs exert their function. Through bioinformatic analysis with online platforms, miR-765 was identified as a potential target of lncRNA RP11-465B22.8. miRNAs are a group of small, well-conserved, noncoding RNAs that regulate the expression of mRNAs [17]. miRNAs are key regulators in many biological and pathological processes, such as cell differentiation, apoptosis, proliferation, migration, and metabolism [17]. Therefore, their translational application as diagnostic markers or potential therapeutic targets for disease treatment has been extensively studied [18]. miR-765 has been reported to function as a tumor suppressor in a variety of cancers, such as pancreatic cancer, ovarian cancer, colorectal cancer, gastric cancer, and renal cell carcinoma [19–23]. In this study, we found that miR-765 was a target of lncRNA RP11-465B22.8. Reinforce of miR-675 abolished the promoting effects of lncRNA RP11-465B22.8 on cell proliferation, migration, and tube formation. Our data suggest that lncRNA RP11-465B22.8 can act as a ceRNA for miR-765 in EC.

KLK4 is a serine protease that belongs to kallikrein-related peptidase, which contains 15 members [14]. Increasing studies have suggested that KLK4 plays a tumor-supporting role in cancer. KLK4 has been indicated to be a biomarker of prognosis in triple-negative breast cancer and ovarian cancer [14, 15]. Inhibition of KLK4 suppresses the growth of oral squamous cell carcinoma cells through the Wnt/β-catenin pathway [16]. Downregulation of KLK4 results in decreased migration and invasion in oral squamous cell carcinoma cells [24]. KLK4 is also reported to contribute to nonsmall cell lung cancer progression [25]. However, the function of KLK4 in EC remains unclear. Herein, we found that KLK4 is a direct target of miR-765. Moreover, overexpression of lncRNA RP11-465B22.8 increased the expression of KLK4, which was decreased by transfection of miR-765. Therefore, our data indicated that lncRNA RP11-465B22.8 enhanced EC cell proliferation and migration via miR-765/KLK4 cascade.

Macrophages are crucial components in the tumor environment, which are important regulators in tumorigenesis and metastasis [26]. Macrophages can be classed into two phenotypes, the proinflammatory M1 and anti-inflammatory M2 macrophages [26, 27]. M1 macrophages can phagocytose cancer cells, while M2 macrophages such as tumor-associated macrophages promote tumor cell growth, migration, and invasion [27]. Macrophages infiltrate tumor tissues and gain a polarized M2 phenotype driven by tumor-derived or T cell-derived cytokines/molecules [28]. It has been shown that tumor cells can cross-talk with immune cells by secreting exosomes, thereby inducing immune suppression [29]. A number of exosomal lncRNAs have been reported to be involved in cell-cell communication and are highly associated with macrophage polarization [30–32]. Consistently, in this study, we found that exosomal lncRNA RP11-465B22.8 was an inducer of M2 macrophage polarization. Treatment with exosomes derived from si-RP11-465B22.8/KYSE-150 cells could reduce M2 macrophage-induced migration.

In conclusion, in the present study, we found the expression of lncRNA RP11-465B22.8 was increased in EC tissues and high lncRNA RP11-465B22.8 expression was linked to poor survival of EC patients. LncRNA RP11-465B22.8 promoted the proliferation, migration, and invasion of EC cells via miR-765/KLK4 axis. Moreover, our data revealed that exosomal lncRNA RP11-465B22.8 could induce M2 macrophage polarization and thus enhance EC cell motility (Fig. 7D). Therefore, our findings provide evidence that lncRNA RP11-465B22.8 is an oncogene in EC, which may serve as a potential target for EC therapy.

## Materials and methods

### Patient samples and ethical statement

EC tissues and their matched adjacent normal tissues were obtained from 26 patients at Xinhua Hospital Affiliated with Shanghai Jiaotong University School of Medicine. Informed consent was obtained from all patients. Patients with other kinds of cancer were excluded from this study. The work was approved by the Ethics Committee of Xinhua Hospital Affiliated with Shanghai Jiaotong University School of Medicine.

### Cell culture and antibodies

EC cell lines TE-1, KYSE-150, KYSE-510, normal HEEC cells, and mononuclear cells THP-1 were purchased from the American Type Culture Collection (ATCC, USA). All cell lines were cultured in RPMI-1640 medium containing 10% (v/v) fetal calf serum (FBS, Gibco, USA) and 1% (v/v) penicillin–streptomycin (Gibco, USA). For macrophage differentiation, THP-1 cells were maintained in a medium containing 100 ng/ml PMA (Sigma, USA) for 48 h. All cell lines had no mycoplasma contamination. Antibodies against CD9 (#13403), CD81 (#10037), CD206 (#24595), Arginase-1 (#93668), E-cadherin (#3195), N-cadherin (#13116), Vimentin (#5741), and GAPDH (#2118) were purchased from Cell Signaling Technology. TSG101 (GTX70255) was purchased from GeneTex.

### Isolation and characterization of exosomes

KYSE-150 cells were transfected with si-RP11-465B22.8. Forty-eight hours after transfection, the culture medium was collected for exosome isolation. To remove residual cells and debris, the culture medium was centrifuged at 300*g* for 10 min, 2000*g* for 10 min, and 10,000*g* for 30 min. Then the supernatant was ultra-centrifuged at 100,000*g* for 70 min to pellet the exosomes. Exosomes were washed once with phosphate-buffered saline (PBS) and harvested by ultra-centrifugation at 100,000*g* for 70 min. The morphology of exosomes was detected by TEM. Briefly, exosomes were resuspended in PBS (Seyotin, China) and dropped on a copper grid. Then, exosomes were fixed with glutaraldehyde, stained with uranyl acetate solution, and photographed.

### The uptake of exosomes

Isolated exosomes were labeled with PKH67 dye using a PKH67 Green Fluorescent Cell Linker Kit (Sigma, USA) following the manufacturer’s protocols. PKH67-labeled exosomes (2 µg) were incubated with THP-1 cells treated with PMA. After incubation for 24 h, PMA-induced macrophages were fixed with paraformaldehyde, stained with DAPI, and visualized under fluorescence microscopy.

### Luciferase reporter assays

HEK293T cells were seeded in six-well plates. To determine the interaction between lncRNA RP11-465B22.8 and miR-765, HEK293T cells were co-transfected with wild-type or mutated lncRNA RP11-465B22.8 and miR-765 mimics (or control miRNA) using Lipofectamine 2000 (Invitrogen, CA, USA). To detect the interaction between miR-765 and KLK4, HEK293T cells were co-transfected with wild-type or mutated KLK4 3′-UTR and miR-765 mimics (or control miRNA). Forty-eight hours post-transfection, cells were collected and the firefly and renilla luciferase activities were determined using a Dual-Luciferase Reporter Assay Kit (Promega, USA).

### Quantitative real real-time PCR (qRT-PCR)

Total RNA was extracted using Trizol reagent (Invitrogen) and reversed transcribed to cDNA by using the SuperScript III cDNA Synthesis kit (Seyotin, China). For miRNA detection, cDNA was synthesized with the TaqMan^™^ Advanced miRNA cDNA Synthesis kit (Thermo Fisher Scientific, USA). Real-time PCR was performed using the Applied Biosystem SYBR (Seyotin, China) according to the manufacturer’s introductions. mRNA expression was normalized to the internal control GAPDH while miRNA expression was normalized to the internal control U6. The relative gene expression was calculated using the 2^−^^ΔΔCT^ formula. All primers are listed in Supplementary Table 1, Primers for miR-765 and U6 were obtained from Ribo (Guangzhou, China).

### Western blot

Samples of cells or exosomes were lysed in radioimmunoprecipitation assay lysis buffer which was supplemented with protease and phosphatase inhibitors (Sigma). The protein sample was separated by SDS-PAGE gels and transferred onto the polyvinylidene fluoride membrane (Millipore). The membrane was blocked with 5% non-fat milk blocking buffer. After incubation with primary antibodies at 4 °C for overnight, the membrane was incubated with horseradish peroxidase-conjugated secondary antibodies at room temperature for 60 min. The protein bands were detected by using the chemiluminescent Western Blotting Substrate (Seyotin, China).

### siRNA transfection

siRNA transfection was performed with Lipofectamine^™^ 2000 reagent (Invitrogen, CA, USA). siRNAs for lncRNA RP11-465B22.8 and KLK4 and control siRNA were synthesized by GenePharma Company (Suzhou, China).

### The migration and invasion assays

Twenty-four-well plates with 8 μm Transwell chambers (Corning, MA, USA) were used for the migration and invasion assays. A total of 1 × 10^5^ cells in 100 μl serum-free medium were seeded in the inserts and 600 μl medium with 10% FBS was added into the lower chamber. For coculture assay, macrophages (1 × 10^4^) in 600 μl medium containing 10% FBS were placed in the lower chamber. After 24 h post-incubation, cells on the top membrane of inserts were scraped by a cotton swab and cells on the bottom of the membrane were stained with 0.5% crystal violet. The number of migrated cells was counted under a light microscope. For the invasion assay, the inserts were coated with Matrigel (BD Bioscience, CA, USA) prior to the assay.

### Immunohistochemistry

Tissue samples were fixed and embedded in paraffin. After dewaxing and dehydration, tissue sections were antigen-retrieved and blocked. Then tissues were incubated with primary antibodies against CD9 or CD163. After washing with PBS, the samples were incubated with a biotinylated secondary antibody, followed by incubation with the horseradish peroxidase–streptavidin reagent (Santa Cruz Biotechnology). Tissue sections were detected with diaminobenzidine solution with hematoxylin counterstaining.

### Animal experiments

BALB/c nude mice (male, 4–6-weeks-old) were obtained from the Institute of Zoology, Chinese Academy of Sciences, Beijing, China. The mice were randomly divided into two groups and luciferase-labeled TE-1/RP11-465B22.8 or luciferase-labeled control cells were injected into the oxter of nude mice (*n* = 5). After 4 weeks, luciferase intensity was monitored and mice were sacrificed. Tumors were isolated and photographed. For in vivo metastasis assay, TE-1/RP11-465B22.8 or control cells were injected into mice via tail vein (*n* = 3). Five weeks later, mice were sacrificed and lung tissues were extracted. The metastatic node in lung tissues was counted and the histological changes of lung tissues were accessed by hematoxylin-eosin staining. All animal experiments were approved by the Institutional Animal Care and Use Committee (IACUC) of Xinhua Hospital Affiliated with Shanghai Jiaotong University School of Medicine.

### Statistical analysis

The sample size in each experiment was chosen to ensure adequate power to detect a pre-specified effect. Studies were not blinded to investigators. Data were expressed as mean ± SD from three independent experiments. Differences between different groups were addressed by the Student’s *t* test or one-way ANOVA analysis. The variance was similar between the groups that were statistically compared. Survival analysis was performed using the Kaplan-Meier method with the log-rank test. A two-tailed value of *P* < *0.05* was considered statistically significant. All statistical analyses were performed with the SPSS 13.0 statistical software (SPSS Inc., Chicago, IL, USA).

## Supplementary information

Supplementary figure legends

Supplementary table1

Supplementary table 2

Figure S1

FigureS2

FigureS3

FigureS4

FigureS5

contribution-form

## Data Availability

For data availability, please contact the corresponding author.
