# Supplementary material for: LncRNA RP11-465B22.8 triggers esophageal cancer progression by targeting miR-765/KLK4 axis
Source: Cell Death Discov. 2021 Sep 24;7:262. doi: 10.1038/s41420-021-00631-9 (PMC8463694; doi:10.1038/s41420-021-00631-9)
Supplement: Supplementary file 1 — Supplementary figure legends [file 41420_2021_631_MOESM1_ESM.doc]

**Supplementary figure legends**

**Figure S1. Overexpression of lncRNA RP11-465B22.8 promotes cell proliferation and induces apoptosis in KYSE-510 cells.** A, qRT-PCR analysis of lncRNA RP11-465B22.8 expression in KYSE-510 cells after lncRNA RP11-465B22.8 overexpression. B, CCK-8 assay analysis of the viability of KYSE-510 cells after lncRNA RP11-465B22.8 transfection. C, Apoptosis analysis of KYSE-510 cells following lncRNA RP11-465B22.8 overexpression.

**Figure S2. The expression of apoptosis-related proteins in EC cells after lncRNA RP11-465B22.8 overexpression or knockdown.** The protein expressions of cleaved caspase3, caspase3, BCl-2 and Bax were determined by Western blot. *P < 0.05, **P <0.01.

**Figure S3. miR-765 inhibits EC cell viability.** A, qRT-PCR analysis of the expression of miR-765 in EC cells after transfection with miR-765 mimics. B, The cell viability of ECs after transfection with miR-765 mimics determined by CCK-8. *P < 0.05.

**Figure S4. miR-765 inhibits EC cell migration.** Transwell migration and invasion assays were used to detect the effects of miR-765 on EC cell migration and invasion. *P < 0.05, **P <0.01.

**Figure S5. miR-765 increases E-cadherin expression EC cells.** Immunofluorescence staining was used to detect the expression of E-cadherin in EC cells after transfected with miR-765 mimics. DAPI was used to stain the nucleus. **P <0.01.

**Supplementary table1** Primer sequences used for reverse transcription-quantitative PCR.

**Supplementary table2** Association between lncRNA RP11-465B22.8 expression in EC tissues and clinical features of patients
