# Supplementary material for: LncRNA RP11-465B22.8 triggers esophageal cancer progression by targeting miR-765/KLK4 axis
Source: Cell Death Discov. 2021 Sep 24;7:262. doi: 10.1038/s41420-021-00631-9 (PMC8463694; doi:10.1038/s41420-021-00631-9)
Supplement: Supplementary file 2 — Supplementary table1 [file 41420_2021_631_MOESM2_ESM.doc]

Table 1. Primer sequences used for reverse transcription-quantitative PCR.

| Genes | Primer sequence (5’🡪3’) |
| --- | --- |
| CD163 | F: TTTGTCAACTTGAGTCCCTTCAC |
|  | R: TCCCGCTACACTTGTTTTCACC |
| Arginase-1 | F: GGTTTTTGTTGTTGCGGTGTTC |
|  | R: CTGGGATACTGATGGTGGGATGT |
| CD206 | F: GGGTTGCTATCACTCTCTATGC |
|  | R: TTTCTTGTCTGTTGCCGTAGTT |
| KLK4 | F:CGGCACCCAGAGTACAACAG |
|  | R: GAAACGAGGCAAGAGTTCCCC |
| RP11-465B22.8 | F: GTACCCAGAGCCTCCCTGTG |
|  | R: GGTGCGTGAACTGCAGACTT |
| β-actin | F: AAGAGCTACGAGCTGCCTGA |
|  | R: GACAGCACTGTGTTGGCGTA |
