# Supplementary material for: LncRNA RP11-465B22.8 triggers esophageal cancer progression by targeting miR-765/KLK4 axis
Source: Cell Death Discov. 2021 Sep 24;7:262. doi: 10.1038/s41420-021-00631-9 (PMC8463694; doi:10.1038/s41420-021-00631-9)
Supplement: Supplementary file 3 — Supplementary table 2 [file 41420_2021_631_MOESM3_ESM.doc]

Table 2 Association between lncRNA RP11-465B22.8 expression in EC tissues and clinical features of patients

| Clinicopathological | case | LncRNA RP11-465B22.8 expression | |
| --- | --- | --- | --- |
|  |  | High | Low |
| Ages |  |  |  |
| 60 or Less | 11 | 6 | 5 |
| >60 | 15 | 8 | 7 |
| Gender |  |  |  |
| Males | 22 | 12 | 10 |
| Females | 4 | 3 | 1 |
| TNM staging |  |  |  |
| Ⅰ/Ⅱ | 11 | 3 | 8 |
| Ⅲ/Ⅳ | 15 | 11 | 4 |
| lymph node metastasis |  |  |  |
| No | 10 | 3 | 7 |
| N1 or more | 16 | 10 | 6 |
|  |  |  |  |
