# Supplementary figures and images for: LncRNA RP11-465B22.8 triggers esophageal cancer progression by targeting miR-765/KLK4 axis
Source: Cell Death Discov. 2021 Sep 24;7:262. doi: 10.1038/s41420-021-00631-9 (PMC8463694; doi:10.1038/s41420-021-00631-9)

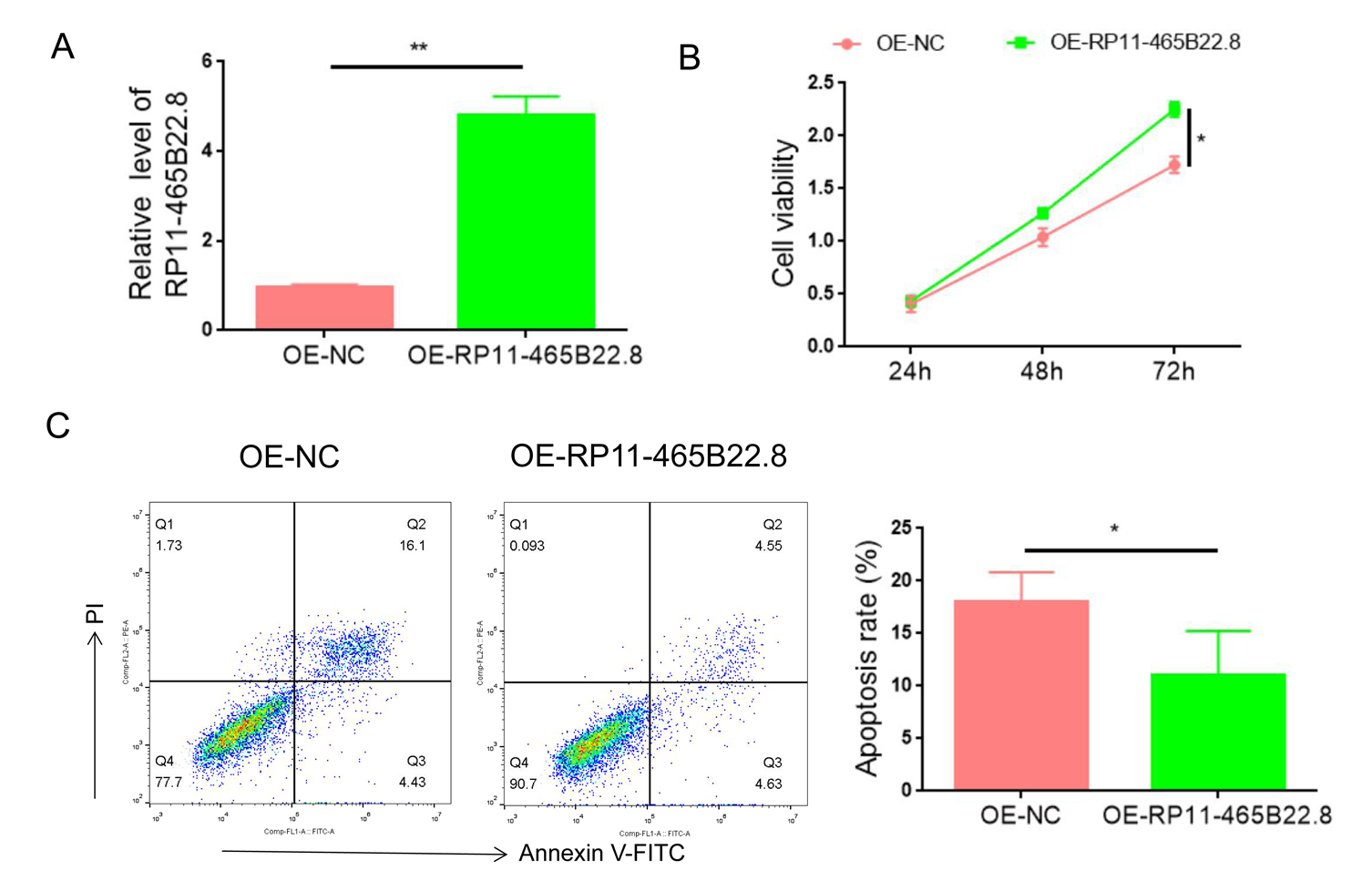

Supplement: Supplementary file 4 — Figure S1 [file 41420_2021_631_MOESM4_ESM.tif]

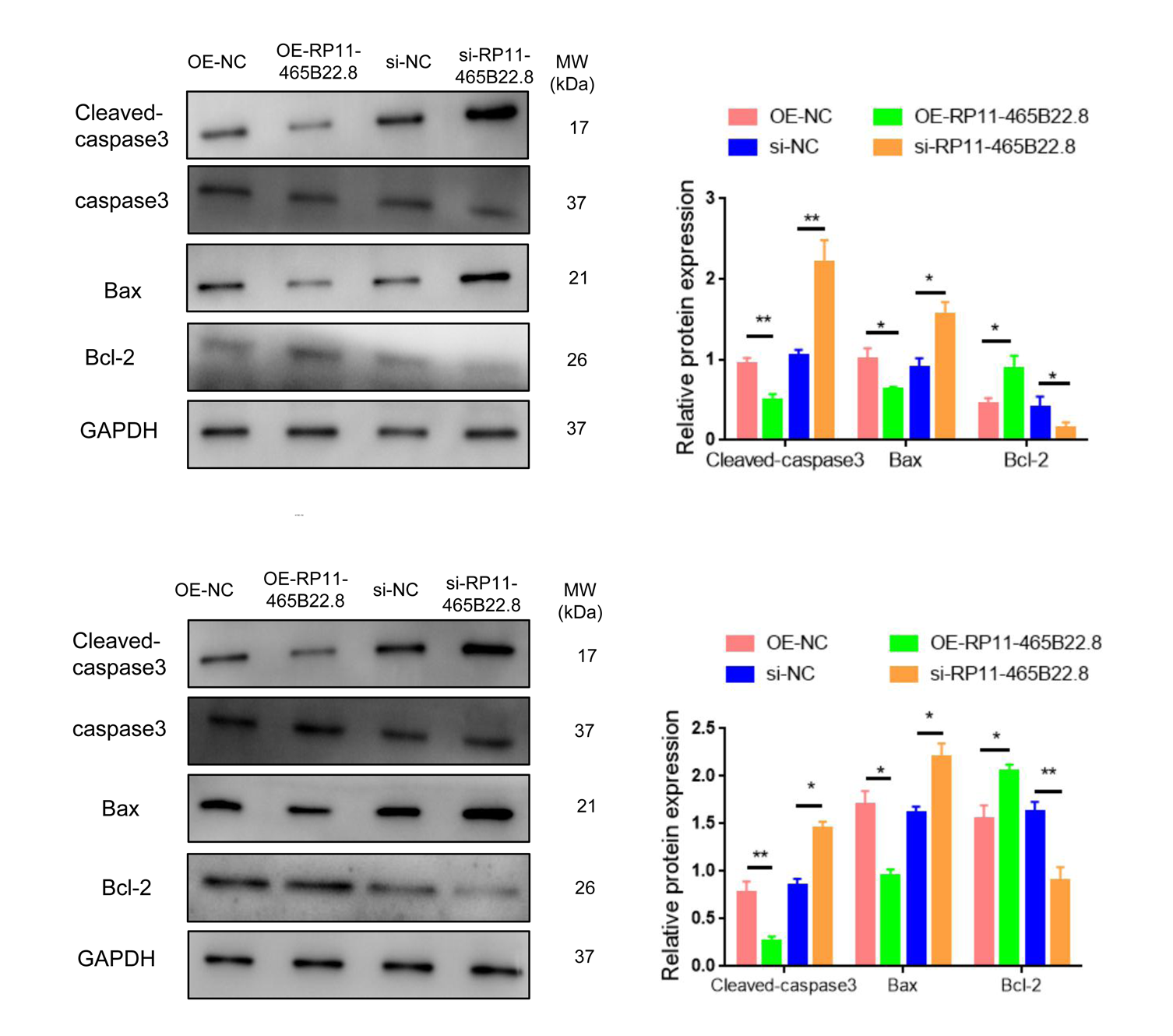

Supplement: Supplementary file 5 — FigureS2 [file 41420_2021_631_MOESM5_ESM.tif]

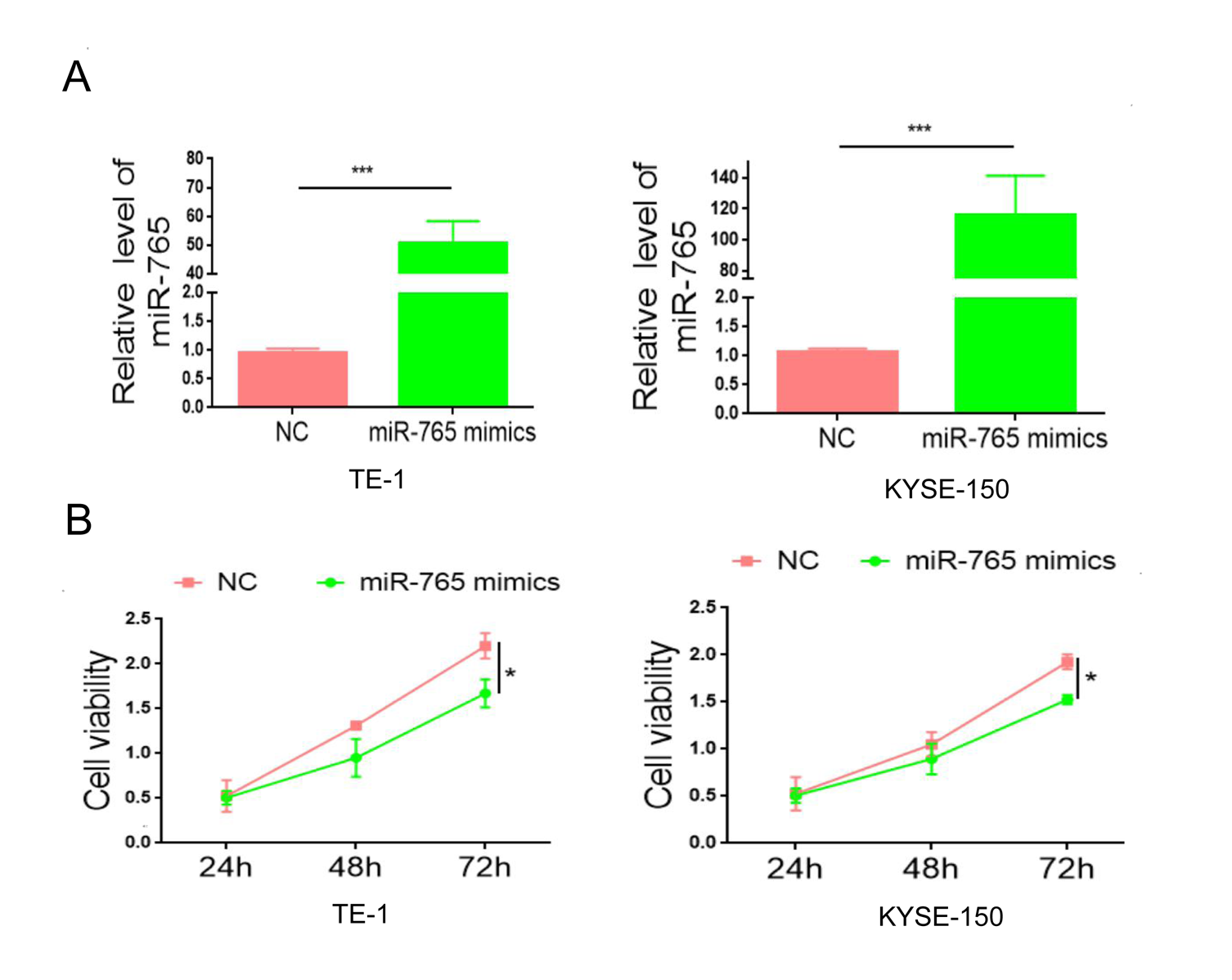

Supplement: Supplementary file 6 — FigureS3 [file 41420_2021_631_MOESM6_ESM.tif]

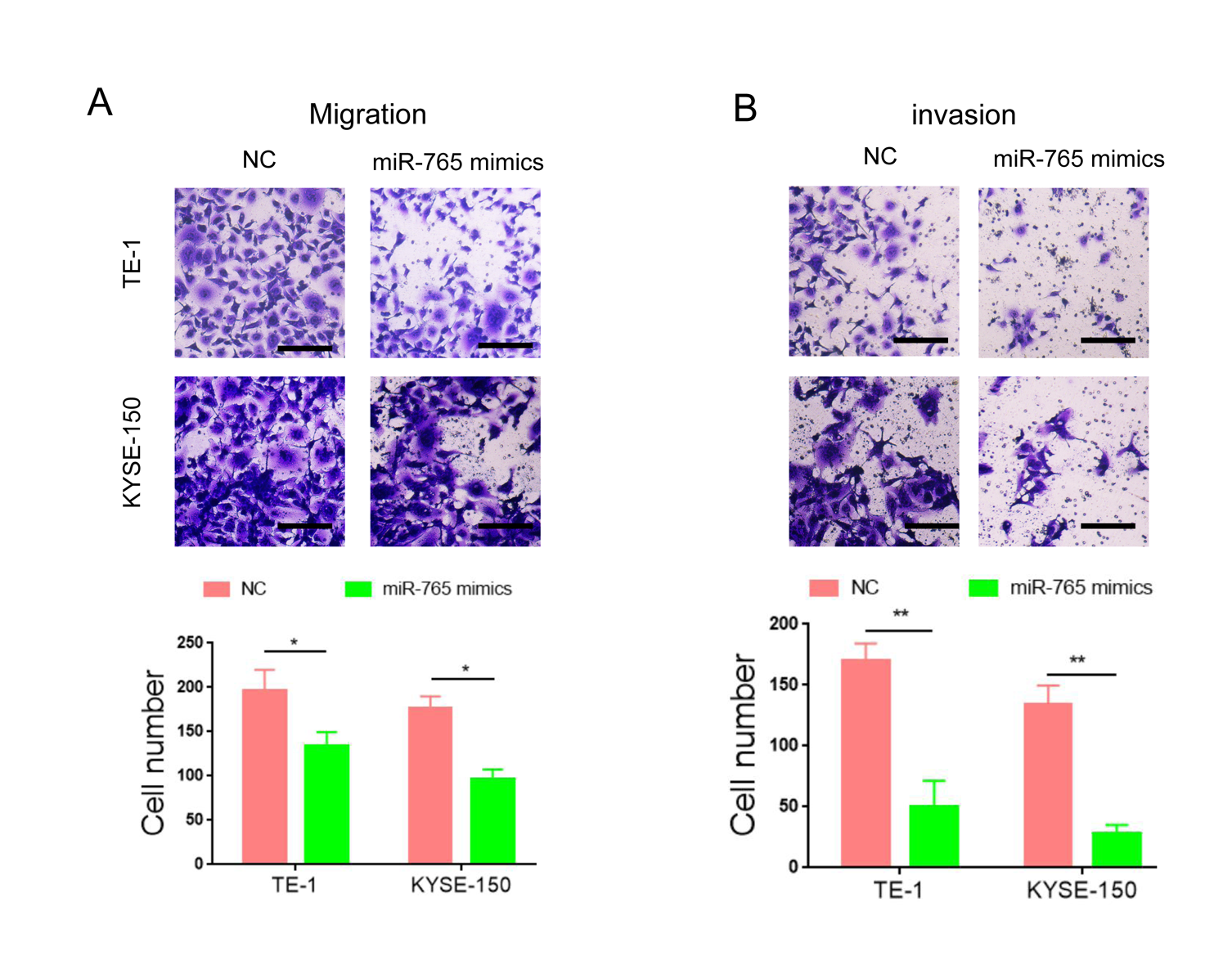

Supplement: Supplementary file 7 — FigureS4 [file 41420_2021_631_MOESM7_ESM.tif]

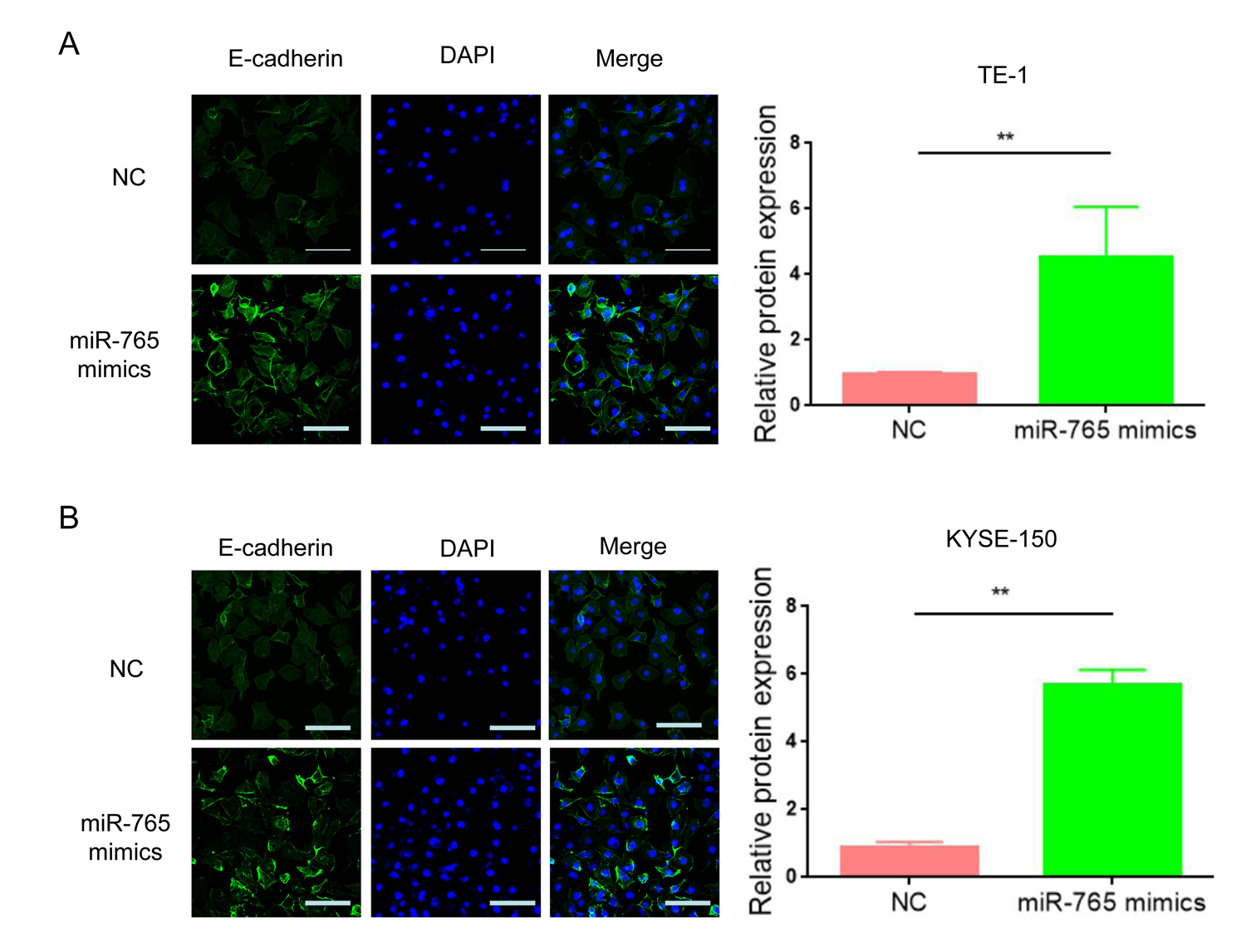

Supplement: Supplementary file 8 — FigureS5 [file 41420_2021_631_MOESM8_ESM.tif]
