# Supplementary material for: LncRNA RP11-465B22.8 triggers esophageal cancer progression by targeting miR-765/KLK4 axis
Source: Cell Death Discov. 2021 Sep 24;7:262. doi: 10.1038/s41420-021-00631-9 (PMC8463694; doi:10.1038/s41420-021-00631-9)
Supplement: Supplementary file 9 — contribution-form [file 41420_2021_631_MOESM9_ESM.pdf]

DECLARATION OF CONTRIBUTIONS TO ARTICLE **ADMC**

Manuscript Number: CDDISCOVERY-21-1550R

Journal Name: *Cell Death Discovery* (the 'Journal')

Proposed Title of the Contribution: LncRNA RP11-465B22.8 triggers esophageal cancer progression by targeting miR-765/KLK4 axis (the 'Contribution)

Author(s): Rui Hu, Rui Bi, Lianyong Jiang, Haibo Xiao, Xiao Xie, Hongtao Liu, Fengqing Hu (the 'Authors')

For all *CDDis*covery articles, each person named as an author in the published version must be able to show he or she has contributed substantially to the article.

Authorship credit should be based on 1) substantial contributions to conception and design, acquisition of data, or analysis and interpretation of data; 2) drafting the article or revising it critically for important intellectual content; and 3) final approval of the version to be published. Authors should meet conditions 1, 2 and 3.

Any person who cannot be shown to have made a substantial contribution to the article cannot be listed as an author in the final version. The name of any person who is deemed to have made a minor contribution can, however, appear in the Acknowledgments section of the article.

Please complete the table below to indicate the contributions of all named authors to the manuscript.

Author Full Name:	Specification of Contribution to the Manuscript:
Rui Hu	Conception and design
Rui Bi	Collection and assembly of data
Lianyong Jiang	Manuscript writing
Haibo Xiao	Data analysis and interpretation
Xiao Xie	Data analysis and interpretation
Hongtao Liu	Data analysis and interpretation
Fengqing Hu	revising it critically for important intellectual content

Please complete the table below to indicate the contributions of all named authors to the figures.

Figure 1:
Rui Hu, Rui Bi

Figure 2:
Lianyong Jiang, Rui Hu, Rui Bi

Figure 3:
Haibo Xiao, Rui Hu, Rui Bi

Figure 4:
Xiao Xie, Rui Hu, Rui Bi

Figure 5:
Hongtao Liu, Rui Hu, Rui Bi

Figure 6:
Fengqing Hu, Rui Hu, Rui Bi

Signed for and on behalf of the Author(s):	Print Name:	Date:
Fengqing Hu	Fengqing Hu	2021.7.12.
